# Family caregiver role in care recipient preparedness for hospital discharge: A parallel mediation model

**DOI:** 10.1371/journal.pone.0327758

**Published:** 2025-07-17

**Authors:** Nosaiba Rayan-Gharra, Orly Tonkikh, Nurit Gur-Yaish

**Affiliations:** 1 The Cheryl Spencer Department of Nursing, Faculty of Social Welfare and Health Sciences, University of Haifa, Haifa, Israel; 2 Oranim Academic College of Education, Tivon, Israel; STIKES Wira Medika PPNI Bali: Sekolah Tinggi Ilmu Kesehatan Wira Medika PPNI Bali, INDONESIA

## Abstract

**Background:**

Family members or friends (caregivers) play a substantial role in supporting health needs of older adults or persons with chronic conditions and accompanying them in acute-care settings. Scarce evidence on the impacts of caregiver engagement in ensuring and explaining medical care during hospitalization calls for further exploration of the role of caregivers along the peri-hospitalization trajectory in supporting hospitalization outcomes. This study aimed to examine the association between pre-admission caregiver support and care recipients’ preparedness for discharge and whether caregiver engagement during hospitalization and upon discharge mediates this association.

**Methods:**

Secondary analysis of a cohort study of 443 internal medicine patients who were accompanied by caregivers. Pre-admission caregiver support, engagement in ensuring and explaining medical care during hospitalization, and covariates like health literacy, demographics and health status were reported by patients during hospitalization. Caregiver involvement in the discharge briefing and care-transition preparedness was reported a week after discharge.

**Results:**

Multivariate mediation analysis showed significant direct effect (B_(unstandardized)_=1.55; CI = 0.09–2.99; *P* = 0.036) of pre-admission caregiver support on care-transition preparedness. The effect was mediated by high caregiver engagement in ensuring and explaining medical care during current hospitalization (Mediated effect (ME)=2.84; CI = 1.78–4.00) and involvement in the discharge briefing (ME = 0.71; CI = 0.20–1.29), controlling for patient and caregiver health and functional status, health literacy, and demographics (Total effect: B_(unstandardized)_=5.10; CI = 3.21 to 7.01). Of the covariates, only high health literacy levels of the patients (B_(unstandardized)_=4.56; *P* < .001) and of the caregivers (B_(unstandardized)_=6.67; *P* < .001) were positively associated with preparedness for discharge.

**Conclusions:**

This study highlights the critical role of caregiver engagement during the hospitalization process as a pathway linking pre-admission support to discharge preparedness. The findings support early identification of caregiving status and health literacy at admission, enabling healthcare providers to tailor communication and better engage caregivers in the discharge process, to ultimately promote smooth care transitions.

## Introduction

Hospital discharge is a particularly vulnerable period in the care trajectory and has been linked to unwarranted hospitalization outcome [1]. Many transitional care-related adverse events result from breakdowns in communication between care recipients (patients) and providers [2]. Patients are often the only link between hospital and community care, and they need to follow many instructions for medications, tests, and referrals [3–6]. Transitional care preparedness, or the extent to which patients report that they know the care plan, the purpose and side effects of each medication, and how to detect warning signs, is associated with hospitalization outcomes and is thus considered an indicator of the quality of the transition from hospital to home [7]. High patient perception of preparedness for transition is associated with better health outcomes [8,9]. By contrast, low patient perception of preparedness for transition and failure in transitional care result in a wide range of adverse outcomes, such as medical and medication errors and excessive resource use, including higher risk for readmission and emergency department visits [1,10,11].

Family members and friends—unpaid family caregivers (caregivers)—play a substantial role in supporting function and other clinical and mental needs of older persons or persons with chronic conditions [12,13]. At home, caregivers provide, manage, and coordinate medical and nursing care tasks, help with transportation, and advocate for the care recipient [12]. Over time, they accumulate knowledge and experience [14,15]. Therefore, engaging them as part of the healthcare team across different healthcare settings is imperative.

Caregivers usually accompany patients in acute care settings [13]. They frequently play an instrumental role in coordinating and managing patient care during hospitalization [16–18] and during transition from the hospital [19,20]. The presence of caregivers and their communication with nursing staff regarding the patient’s condition throughout hospitalization are considered essential for effective information sharing, patient safety, and the provision of holistic and continuous care [21–23]. The presence of a caregiver is particularly important during the discharge process. Their capacity to obtain and understand basic health information and their ability to make appropriate health decisions plays a substantial role in high patient preparedness for transition [24,25], beyond patient complexity [26] and prior admissions [24]. Accordingly, recent initiatives, such as the Caregiver Advise, Record, Enable (CARE) Act, encourage identifying caregivers upon admission and providing them education and instruction on the medical-related care responsibilities needed at home [27–29].

Identifying caregivers upon admission provides an opportunity to work with them during hospitalization to maximize their engagement, beyond the discharge process. Asking questions, holding discussions with the care recipient and staff during hospitalization, might be an opportunity to process knowledge required to support post-discharge care. Scarce evidence on the impacts of caregiver engagement in ensuring and explaining medical care during hospitalization calls for further exploration of the role of caregivers along the peri-hospitalization trajectory in supporting hospitalization outcomes.

This study is guided by the Family Involvement in Interpersonal Health Care Processes Framework [30], which emphasizes the role of family factors in shaping communication and coordination of care through their involvement in medical encounters. Caregivers play a central role in information exchange, helping patients understand medical instructions, clarifying needs, and ensuring information is accurately transferred between patients and providers. These caregiver contributions, especially in high-risk environments like hospital discharge planning, have been associated with improved care coordination, quality of care and patient outcomes. We conceptualize pre-admission caregiver support as a family attribute that is associated with caregiver engagement during hospitalization and discharge, which in turn contributes to care-transition preparedness. Based on this framework, the current study examines: (a) the association between pre-admission caregiver support and patient preparedness for discharge from hospital to home and (b) whether caregiver engagement in ensuring and explaining care during hospitalization and upon discharge mediates this association.

## Materials and methods

### Design, participants and setting

We conducted a retrospective observational study (secondary data analysis, accessed in October, 2022) using data from a prospective cohort study designed to examine cultural factors, health practices, and hospitalization outcomes [31]. Inclusion criteria were community-dwelling adults (aged 18+) hospitalized for an unplanned admission of at least one night who were able to complete a survey in Hebrew, Arabic, or Russian and who were members of Clalit Health Services (the largest not-for-profit integrated healthcare provider and insurer in Israel). Over-sampling of the Arab-speaking and Russian-speaking populations was performed to enable a large-enough sample for testing care-transition preparedness among vulnerable populations. Exclusion criteria were a diagnosis of Alzheimer’s disease and related dementia, diagnosis of acquired immunodeficiency syndrome, receiving palliative or end-of-life care, a do-not-resuscitate directive, and lacking an active telephone number for follow-up. The recruitment process is fully described elsewhere [31].

### Data collection

The population of the original cohort study included 675 adults admitted through the emergency department to internal medicine units in a tertiary medical center in Israel in 2014. The patients were asked to report caregiver peri-hospitalization engagement and preparedness for discharge using validated questionnaires during and after the hospitalization. Data on clinical status, length of stay and prior hospitalizations were retrieved during 2016 from Clalit’s data warehouse, the largest health maintenance organization (HMO) in Israel, and were merged with the survey data. Of the 675 participants recruited to the study, a subset of 443 (69.0%) patients who were accompanied by a caregiver and completed in-hospital and post-discharge questionnaires were used for the current investigation. Of those, 24 patients accompanied by a paid caregiver and one participant with missing information on pre-admission caregiver support were excluded. During hospitalization (at least the second day of admission), participants were asked to report about ensuring and explaining in-hospital care provided by their caregiver during the current hospitalization and the caregiver support before the current hospitalization. Participants were also asked to report their sociodemographic and cultural characteristics, health status, health literacy, native language, education level, age, gender, and economic status. In addition, they were asked to report their relationship with the caregiver as well as the caregiver health literacy, health status, age, and sex. Three to seven days after discharge, patients were surveyed by phone about caregiver involvement in the discharge process and about the transition preparedness from hospital to home. Questionnaires were administered in Hebrew, Arabic, and Russian.

### Measures

#### Outcome measure.

Care-transition-preparedness was measured using the adapted 12-item care transition measure (CTM) for use in Israel [24] to assess patients’ care-transition experience and the extent to which patients were prepared for discharge from hospital to home (preparedness for hospital discharge) [7,32]. Answers are rated on a 4-point scale ranging from “strongly disagree” to “strongly agree.” Higher scores on the 0–100 CTM scale represent better care-transition preparedness (Cronbach’s alpha = 0.93).

#### Mediators.

Engagement in ensuring and explaining in-hospital care (Mediator 1) was measured with the Ensuring and Explaining subscale of the Informal Assistance and Support for Hospitalized Older Adults scale (ICHOA) [16]. The three-item Ensuring and explaining subscale addresses the frequency of help that the respondent (the patient) received from the caregiver during their hospitalization with the following: “keeping an eye on care”, “discussing condition with staff”, and “helping to understand the condition”. The response scale was a Likert scale (1 = did not receive any help; 5 = received help all the time; Cronbach’s alpha = 0.90). Caregiver involvement in the discharge briefing (Mediator 2) was measured using a modified version of the Quality of Discharge Briefing index [33] using the following binary (0 “no”, 1 “yes”) items: “Was a family caregiver present at the time you received the discharge instructions?” and “Did a family caregiver discuss the discharge recommendations with the healthcare team?” A summed score was computed.

#### Independent variable.

Pre-admission caregiver support: Caregiver support prior to the current hospitalization was assessed with the Functional Solidarity dimension of the Longitudinal Study of Generations tool [34]. The intensity of assistance with personal care/activities of daily living, household chores, transport and shopping, and emotional support was measured on a 5-point Likert scale (1 = not at all; 5 = very much). We added three items assessing the caregiver’s previous assistance with medical/nursing care, supervision of performance of medical/nursing tasks [35], and involvement in prior hospitalizations. The internal reliability was estimated using Cronbach’s alpha (0.83).

#### Control variables.

Patient characteristics included health status, assessed using the SF-12 V.2 [36]. Higher scores on the 0–100 (Physical Component Score (PCS) and Mental Component Score (MCS)) subscales represent better physical/mental health. Daily functioning was assessed using the six-item Katz Index of Independence in Activities of Daily Living (ADL) scale [37], with each activity scored as 0 (independent), 1 (partially dependent), or 2 (fully dependent). Scores were summed to produce a total ranging from 0 (independent) to 12 (dependent). Health literacy was measured using the seven-item Brief Health Literacy Screen (BHLS), answered on a 5-point Likert-type scale (1 = never, 5 = always) [38]. The items were summed and dichotomized as low (total score ≤21) or medium–high (total score >21) health literacy [39,40]. Data on length of stay (LOS), number of chronic conditions, and number of hospitalizations in the year prior to the current hospitalization were retrieved from Clalit’s Health Services’ data warehouse. Age, sex, and minority status (Hebrew speakers, Arabic speakers, Russian speakers) were included as control variables.

Family caregiver characteristics, reported by the patient, were also used as control variables and included: health status (assessed using the SF-12 general health item) [41]; health literacy (measured using the three-item BHLS questionnaire) [38]; relationship to the care recipient (spouse, child, other); and caregiver age and sex.

### Ethics approval and consent to participate

The participants recruited to the original study provided written informed consent prior to their participation. The original study, including all its components, was approved by the Ethics Committee of the Faculty of Social Welfare & Health Sciences at the University of Haifa, Israel (Ref No.: 138/May-2014). Specifically, data collection conducted at the hospital was approved by the Institutional Review Board of the Medical Center (Ref No.: 0184-May-2013-RMC), and the approval for data retrieval was granted by Clalit Health Services (Ref No.: 0180-December-2015-COM2).

### Data analysis

Descriptive characteristics of the sample were computed using means and standard deviations for continuous variables and frequencies for categorical variables. The association between pre-admission caregiver support, engagement in ensuring and explaining in-hospital care, and involvement in the discharge process during current hospitalization and its association with patients’ care-transition-preparedness was evaluated using a parallel mediation model. A parallel mediation model was selected, as the two forms of caregiver engagement represent distinct yest concurrent processes that may independently mediate the effect of pre-admission support. Analyses were performed using IBM SPSS statistical package V.27.0 and the PROCESS macro (V.3.4), model 4.

## Results

The sample included 443 adults accompanied by a spouse (*n* = 227, 51.2%), child (*n* = 157, 35.4%), parent (*n* = 27, 6.1%), sibling (*n* = 14, 3.2%), grandchild (*n* = 13, 2.9%), or friend (*n* = 5, 1.1%). Table 1 summarizes the baseline characteristics of the patients and the caregivers accompanying them during hospitalization. The mean care-transition preparedness score was medium (60.8, SD = 16.7). Two hundred thirty-one (52.1%) patients reported frequently (always or almost always) receiving support with ensuring and explaining in-hospital care during hospitalization, and 244 (55.1%) reported caregiver involvement in the discharge briefing. Two hundred seventy-five patients (62.1%) reported intensive pre-admission caregiver support.

**Table 1:** Patients’ and caregiver^*^s’ baseline characteristics (*n* = 443).

Patient characteristics	
Age, y (18–90), mean ± SD	61.8 ± 17.3
Male, *n* (%)	217 (49.0)
High health literacy, *n* (%)	185 (41.8)
Minority, *n* (%)	
Hebrew speakers (reference)	87 (19.6)
Arabic speakers	191 (43.1)
Russian speakers	165 (37.2)
Activities of Daily Living (0–12), mean ± SD	1.7 ± 2.8
Physical Component Score (SF-12) (0–100), mean ± SD	37.8 ± 13.0
Mental Component Score (SF-12) (0–100), mean ± SD	34.3 ± 10.6
Number of diagnoses (0–12), mean ± SD	3.6 ± 2.3
Prior hospitalizations (0–9), mean ± SD	1.2 ± 1.7
Length of stay (1–26), mean ± SD	4.6 ± 3.7
**Caregiver characteristics**	
Age, y (16–92), mean ± SD	50.9 ± 14.9
Male, *n* (%)	114 (25.7)
Relationship with the patient, *n* (%)	
Spouse	227 (51.2)
Child	157 (35.4)
Other	59 (13.4)
High health literacy, n (%)	249 (56.2)
Health status (SF-1) (1–5), mean ± SD	3.6 ± 0.8

* Family member or friend

**Fig 1:**
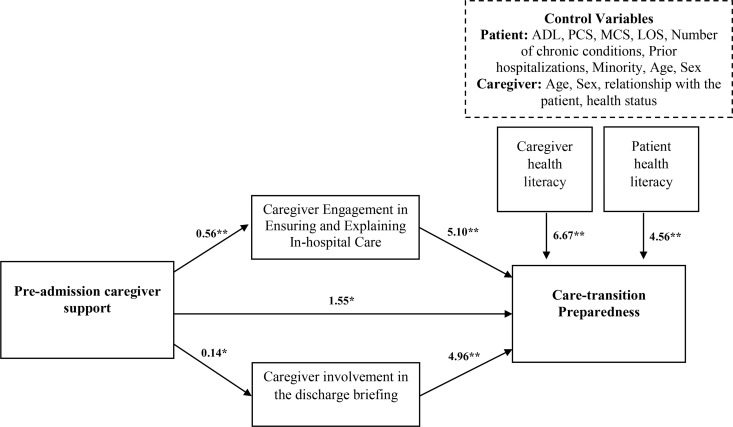
Results for the mediation effect of prior health care experience on preparedness for discharge. The model shows significant *unstandardized* regression coefficients (bold lines). *Dashed lines* highlight *non-significant* relationships of the control variables. *p < 0.01, **p < 0.001. ADL, Activities of Daily Living; MCS, Mental Component Score; PCS, Physical Component Score; LOS, length of stay.

Multivariate mediation analysis showed a significant direct effect (B_(unstandardized)_ = 1.55; CI = 0.09 to 2.99; p = 0.036) and indirect effects (total indirect effect: B = 3.55, CI = 2.30 to 4.89) of pre-admission caregiver support on preparedness for discharge through high engagement in ensuring and explaining in-hospital care during the current hospitalization (mediated effect: B = 2.84; CI = 1.78 to 4.00) and involvement in the discharge briefing (mediated effect: B = 0.71; CI = 0.20 to 1.29), controlling for patient’s functional, mental, physical, and clinical health status; number of prior admissions; and caregiver health status, health literacy, age, and sex (total effect: B = 5.10; CI = 3.21 to 7.01) (Fig 1). A specific indirect-effect contrast (B = 2.12; CI = 0.99 to 3.36) shows that the indirect effect of pre-admission caregiver support on preparedness for discharge through engagement in ensuring and explaining in-hospital care is significantly higher than the indirect effect through and involvement in the discharge briefing. Only the high health-literacy levels of both patients (B = 4.56, CI = 2.12 to 7.00; *P* < .001) and caregivers (B = 6.67, CI = 3.01 to 10.32; *P* < .001) were positively associated with preparedness for discharge. Table 2 lists the unstandardized and standardized regression coefficients.

**Table 2:** Unstandardized and standardized regression coefficients.

	Unstandardized B (SE)	Standardized Beta	*p*-value
Peri-hospitalization caregiver* **engagement**			
Pre-admission caregiver support (independent variable)	1.55 (0.74)	0.08	0.04
Caregiver Engagement in Ensuring and Explaining In-hospital Care (Mediator 1)	5.10 (0.69)	0.42	<.001
Caregiver involvement in the discharge briefing (Mediator 2)	4.96 (0.63)	0.28	<.001
**Patient characteristics**			
Age, y (18–90)	−0.02 (0.05)	−0.02	0.69
Male	−0.99 (1.32)	−0.03	0.45
High health literacy	4.56 (1.24)	0.14	<.001
Minority (Arabic or Russian speaker)	0.53 (1.59)	0.01	0.74
Activities of Daily Living (0–12)	−0.35 (0.23)	−0.06	0.12
Physical Component Score (0–100)	0.003 (0.05)	0.002	0.94
Mental Component Score (0–100)	0.04 (0.06)	0.03	0.48
Number of diagnoses (0–12)	0.29 (0.28)	0.04	0.29
Prior hospitalizations (0–9)	0.23 (0.35)	0.02	0.051
Length of stay (1–26)	−0.0004	0.000	1.00
**Caregiver characteristics**			
Age, y (16–92)	−0.01 (0.06)	−0.01	0.85
Male	0.01 (1.39)	0.00	1.00
Relationship with the patient			
Spouse	1.63 (1.82)	0.05	0.37
Child	0.14 (2.27)	0.004	0.95
High health literacy	6.67 (1.86)	0.20	<.001
Health status (SF-1) (1–5)	−0.88 (0.83)	−0.04	0.29

* Family member or friend

The results suggest that when caregivers provided more support prior to hospitalization, patients felt more prepared for discharge. This relationship was mediated by caregiver engagement during the hospital stay and in the discharge process. Both types of engagement contributed to better preparedness for discharge, with in-hospital engagement having a higher effect than involvement in the discharge briefing. Additionally, higher health literacy levels of both patients and caregivers were associated with better preparedness for discharge.

## Discussion

This study is the first to examine the association between pre-admission caregiver support and engagement in ensuring and explaining in-hospital care during an acute hospitalization and, in turn, patients’ preparedness for discharge. Caregivers who were more involved in emotional support, assistance with healthcare needs or household chores prior to hospitalization engaged more in ensuring and explaining in-hospital care during the current hospitalization and in the discharge briefing. Finally, caregiver support prior and during hospitalization and at discharge were related to better patients’ preparedness for discharge.

Our study sheds light on the peri-hospitalization caregiver engagement in the transition of acutely ill adults from general units to home. Caregivers play a significant role in their care recipient’s transition from hospital to home because of their ability to operationalize and execute the discharge plan [28,42]. Studies show an association between caregiver presence and better preparedness for discharge [24] and lower rates of readmission [31]. The absence of a caregiver to assist with discharge and home care is considered a risk factor for readmission [43]. Previous studies that examined transitional care interventions showed how caregiver involvement improved patients’ physical, mental, and cognitive outcomes; contributed to greater patient satisfaction; and improved continuity of care [24,27,28,31,44,45].

Our study extends the understanding of caregiver presence in terms of the timing and scope of their engagement during the hospitalization. Beyond the time of the hospital discharge, we found an association between engagement in ensuring and explaining in-hospital care during the hospitalization and patients’ preparedness for discharge. Previous findings reported inconsistent results about the impact of in-hospital caregiving on patient outcomes. Caregiver presence [46] and engagement [16,47] during an acute hospitalization were negatively associated with older adults’ in-hospital function [46,47] and positively associated with their mental health [48]. Taken together, these studies and the current investigation suggest that the impact of in-hospital caregiver support on patient outcomes may depend on the specific type or nature of the support provided.

The current investigation highlights the benefit that continuous caregiver engagement in ensuring and explaining in-hospital care provides to preparedness for discharge. Previously, primarily qualitative studies shed light on a potential explanation for this association, indicating that caregivers invest considerable effort to identify needs, fill gaps in provision of care, and advocate for patients to maximize their well-being [49,50]. Moreover, caregivers perceive themselves as responsible for protecting the older person and ensuring they received sufficient care [51], as they see themselves as holding personalized knowledge that enables them to identify the severity of exacerbation in a patient’s condition [52]. Our findings extend these qualitative findings by quantifying pre-admission accumulated personalized caregiving knowledge and its use during hospitalization, enabling generalizability to diverse populations. The predominance of the indirect effect of pre-admission caregiver support on preparedness for discharge through engagement in ensuring and explaining, compared with the indirect effect through the caregiver presence and involvement in the discharge briefing, highlights the beneficial impact of caregiver engagement beyond the discharge-briefing episode.

### The role of family caregiver characteristics (Pre-admission caregiver support and health literacy)

Beyond the importance of caregiver support in discharge and during the hospital stay, this investigation demonstrates a significant association between pre-admission caregiver support and patient outcomes at discharge. Pre-admission caregiver experience in direct care, emotional support, and care coordination might provide them with better readiness to assist patients during the hospital stay. Perceived low levels of readiness for caregiving have been previously associated with patient outcomes, such as higher levels of pain, fatigue, depression, and anxiety in home-care settings [53]. Our findings expand the potential implications of caregiver experience to the hospitalization context and outcome, by reframing the trajectory of caregiver support as a continuum beginning before admission [54]. Perceiving hospitalization as a short episode of a continuous family caregiving experience should guide the design of interventions to support caregiver engagement during hospitalization and ultimately improve patient outcomes. To improve hospitalization outcomes, we recommend utilizing a routine family caregivers involvement assessment at admission to identify pre-admission family caregiving patterns. That information will guide nursing decision making engaging with caregivers during the hospitalization to pre-admission caregiving patterns. Caregivers lacking prior experience/involvement might benefit from more guided engagement during the hospital stay to facilitate the achievement of better preparedness for discharge.

This study shows that caregiver health literacy is associated with patients’ preparedness for discharge, beyond caregiver peri-hospitalization involvement. This finding adds to previously reported consistent results on the association between patients’ health literacy and transitional care outcomes [24,31,55–58]. Our study adds to previous literature in presenting the significance of both patient and caregiver health literacy in explaining patients’ preparedness [25]. This association might be rooted in the predominance of Arabic- and Russian-speaking participants in the sample, representing diverse ethnicities. Arab and immigrants from the former Soviet Union in Israel have lower health-literacy scores than the population of long-term Israeli Jews [59].

### Limitations

This study’s findings should be interpreted in light of several limitations. First, the study’s generalizability may be limited due to characteristics of the Israeli society. Nonetheless, results from the original study were shown to have relevance to diverse patient populations [60]. Caregiving patterns found in our sample are consistent with those reported in more recent studies conducted in Israel [61,62]. Additionally, the oversampling of Arab and Former Soviet Union immigrant populations performed in the original study allowed us to have a sufficiently large sample to analyze family caregiving patterns (timing and scope) variabilities among ethnically and racially diverse populations as identified in previous national surveys [12,63]. Second, the original study excluded patients with Alzheimer’s Disease and Related Dementia, whose caregivers might experience higher burden, strain and unwarranted mental health outcomes [64]. Third, the original study was observational and based on patients’ self-reports using validated instruments. Due to the observational design, the mediation model should not be interpreted as establishing causal pathways. Rather, the model reflects theoretically guided associations between key concepts. Finally, the duration of caregiver support was not assessed in the current study but is known to be associated with caregiver preparedness [65].

### Practical implications

Identifying caregiving status and health literacy at admission allows healthcare providers to better tailor caregiver involvement and communication throughout hospitalization. For caregivers with limited experience, additional guidance can help enhance their engagement and improve discharge preparedness. Hospitals should incorporate caregiver engagement into their policies, establish clear communication channels, and provide targeted training to enhance caregivers’ understanding of care. Practices such as identifying a family caregiver early in the admission process, involving them in conversations with staff, and ensuring they receive clear written instructions can be effectively integrated into both in-hospital care and the discharge process.

### Conclusions

This study is the first to examine the role of the trajectory of caregiver involvement throughout the peri-hospitalization period. Our findings reveal that caregivers who were more involved in providing emotional support, assisting with healthcare needs, or managing household chores prior to hospitalization were also more engaged in ensuring and explaining in-hospital care and in the discharge briefing. This engagement was associated with better patients’ preparedness for discharge. These insights highlight the role of caregiver involvement at multiple stages of the care process and underscore the need for policies and practices that tailor support and caregiver engagement. Future studies should focus on the development and implementation of interventions that facilitate tailored caregiver engagement and evaluate their impact on discharge outcomes.
